# Forward modelling of the completeness and preservation of palaeoclimate signals recorded by ice‐marginal moraines

**DOI:** 10.1002/esp.5371

**Published:** 2022-04-24

**Authors:** Ann V. Rowan, David L. Egholm, Chris D. Clark

**Affiliations:** ^1^ Department of Geography University of Sheffield Sheffield UK; ^2^ Department of Geoscience Aarhus University Aarhus Denmark

**Keywords:** glacial landscape evolution, moraine, mountain glaciers, palaeoclimate, Quaternary geology

## Abstract

Glaciers fluctuate in response to climate change and record these changes by building sedimentary landforms, including moraines. Therefore, glacial landscapes are a potentially valuable archive of terrestrial palaeoclimate change. Typically, a cooling climate causes glaciers to expand and a warming climate causes glaciers to shrink. However, the glacier response time and the influence of mountainous topography on glacier dynamics complicates this behaviour, such that moraines are not always a straightforward indicator of glacier change in response to climate change. We used a glacial landscape evolution model to simulate the response of a hypothetical mountain glacier to simple changes in climate and the resulting formation and preservation of moraines. These results show that the rate of climate change relative to the glacier response time determines the geometry, number, and position of moraines. Glaciers can build distinct moraines in the absence of climate change. The distance from the maximum ice extent may not represent the chronological order of moraine formation. Moraines can be preserved after being overrun and eroded by subsequent glaciations, but moraine sequences may also contain gaps that are unidentifiable in the field.

## Graphical abstract

Forward modelling of moraine building and preservation in a synthetic mountain landscape demonstrates how and when palaeoclimate signals are recorded in glacial landscapes. The rate of climate change relative to the glacier response time determines the geometry, number, and position of moraines. The model results indicate (and suggest some solutions to) potential challenges in interpreting moraine sequences that are unlikely to be identified from either field or remote sensing observations.

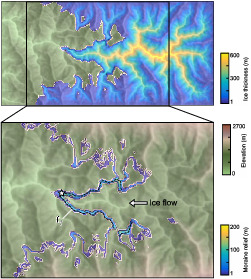

## 1 INTRODUCTION

The transfer of climate signals through the ocean and the atmosphere modifies the behaviour of the climate system from the predictable patterns produced by orbital forcing, and results in variability in the timing and magnitude of climate change between oceanic and terrestrial environments (Broecker, 2003). During periods of abrupt climate change—such as the termination of the Last Glacial Maximum (LGM; Termination 1 occurred around 18 ka)—Earth's climate oscillated rapidly, providing valuable opportunities to understand the transient response of the climate system to internal and external forcing (Clark et al., 2012; Denton et al., 2010). The mechanisms of global climate change can be interpreted from a range of palaeoclimate proxy records (e.g., Overland et al., 2016; Shakun et al., 2012). Although many atmospheric and oceanic palaeoclimate archives are available (e.g., marine sediment cores and ice cores; Kaspari et al., 2008; Lisiecki & Raymo, 2005; Sime et al., 2009) equivalent terrestrial proxies are scarce (e.g., palynological records and lacustrine sediment cores; Bakke et al., 2021; Zhang et al., 2017). Understanding the mechanisms driving the global climate system requires quantifying palaeoclimate at a wide range of latitudes and elevations and across continental land masses, but there are currently few possible terrestrial archives with a wide spatial coverage (Kaufman et al., 2020). Glacial landscapes are one such archive, as glaciers, ice caps and ice sheets fluctuate in response to climatic forcing and record changes in ice volume by building distinctive moraines (Barr & Lovell, 2014; Smedley et al., 2017).

**FIGURE 1:**
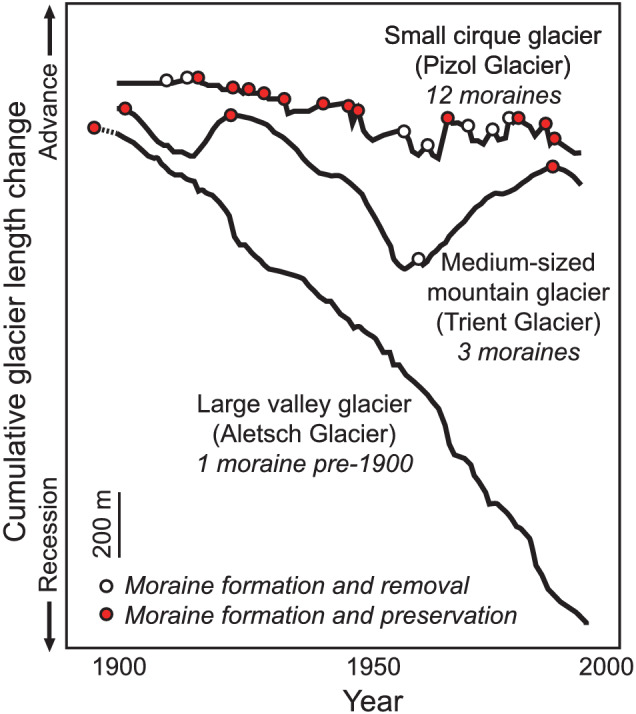
Cumulative length change from observational records for three Swiss glaciers with different dynamic response times during a period of overall recession showing how glacier size affects climatic filtering. Inferred periods of moraine building and preservation are indicated by dots, whereby moraine formation occurs when a glacier advances to a maximum position then recedes, and preservation occurs when not subsequently overrun by a more extensive advance. Small glaciers fluctuate more frequently than larger glaciers and can build a greater number of moraines if sufficient sediment is available (modified from Haeberli, 1995) [Color figure can be viewed at wileyonlinelibrary.com]

Glaciers spread sediment over the landscape forming till sheets and higher‐relief ridges during advance and if the ice margin stabilises during recession (Greenwood & Clark, 2009). These ridges, called ice‐marginal (lateral and terminal) moraines, are observed and preserved in a wide range of terrestrial settings, revealing former glacier dimensions (Penck, 1905). Despite the long‐standing use of ice‐marginal moraines to reconstruct palaeoglaciers and infer palaeoclimate (Mackintosh et al., 2017), to our knowledge, only one study has explored the relationship between glacier expansion and moraine formation (Vacco et al., 2009). While moraines can be a straightforward indicator of glacier length or volume change (e.g., Chandler et al., 2020; Fu & Yi, 2009; Haeberli, 1995) they require careful interpretation because (1) the response of glaciers to climatic forcing depends on non‐linear feedbacks between ice flow and topography (Bahr et al., 1998; Huybers & Roe, 2009) and (2) the preservation potential of these landforms is effectively unknown (Gibbons et al., 1984) (Figure 1).

Statistical models of moraine preservation indicate that the number of moraines observed in a landscape is likely to represent only a third of the number of major glacial advances (Gibbons et al., 1984; Muzikar, 2016). Inferring palaeoclimate from glacier reconstructions requires evaluating past variations in ice extent indicated by moraines to understand the magnitude and timing of ice volume change compared to the present day (e.g., Balco, 2020; Ely et al., 2021; Kelly et al., 2014; Solomina et al., 2015). Palaeoclimate is often inferred by calculating the Equilibrium Line Altitude (ELA) of the reconstructed ice mass to estimate the change in mean annual air temperature (MAAT) from present‐day values represented by the difference in ice volume (e.g., Anderson et al., 2019; Boston et al., 2015; Višnjević et al., 2018). However, this inverse approach is stymied by reliance on the circular reasoning of inferring the local palaeoclimate from moraines that are assumed to be representative of periods of ice margin stability.

The magnitude of glacier length or volume change in response to climatic forcing is strongly influenced by the feedbacks between ice flow and high‐relief topography (Magrani et al., 2021; Pedersen & Egholm, 2013). Therefore, palaeoglacier reconstructions that do not consider these dynamic feedbacks may underestimate or overestimate the climatic forcing suggested by differences in length or volume change between individual glaciers (e.g., Boston et al., 2015; Ely et al., 2021). For example, the distribution of moraine ages produced by the North American ice sheets during the last deglaciation indicate that complex topography underlying the Cordilleran Ice Sheet had a profound influence on ice flow compared to the simple response of the Laurentide Ice Sheet that occupied the lowlands (Menounos et al., 2017). In the European Alps, regional variations in the timing of glacial maxima could have resulted from variations in palaeoclimate, glacier dynamics or uncertainties in the dating methods used to constrain the ages of moraines, or a combination of these factors, which are difficult to explain from inverse modelling (Seguinot et al., 2018). Moreover, the margins of ice sheets and glaciers fluctuate year‐to‐year in response to interannual variability in climate (that is, weather) such that changes in ice margin position of an equivalent magnitude to those observed between the Little Ice Age and the present day may not represent a genuine change in climate (Burke & Roe, 2014; Roe & O'Neal, 2009). Few studies have investigated if interannual fluctuations in ice volume could be recorded by moraine sequences, and if so, how they can be distinguished from moraines formed over longer timescales that are more representative of palaeoclimate change (Anderson et al., 2014; Balco, 2020).

Glacial geomorphology is therefore not yet a reliable indicator of palaeoclimate, and better understanding is required of the climatic–topographic feedbacks and non‐linear glacier behaviours that modify how climate forcing translates into moraine building (Anderson et al., 2012). A tension exists when moraines are used as palaeoclimate indicators because the non‐climatic influences on glacier change need to be resolved (e.g., Chandler et al., 2016; Doughty et al., 2017). Glacier–climate reconstructions rely on the assumption that the moraines remaining after the ice has vanished represent a complete record of glacier change, whereas glaciers can fluctuate without building moraines and erase moraines formed during previous glaciations (Kirkbride & Brazier, 1998). Field observations also demonstrate that moraines can be composite features, where multiple changes in ice volume are superimposed into a single landform (Lukas et al., 2012). Here, we use a novel forward‐modelling approach to test the hypothesis that observable moraine sequences reflect the timing and magnitude of Late Quaternary palaeoclimate change.

## 2 METHODS

We used a glacial landscape evolution model that simulates the erosion, transport and deposition of sediment by glaciers using the higher‐order equations for ice flow (Egholm et al., 2011, 2012) to investigate the impact of climate change on moraine building in a mountainous landscape. The glacier model used a synthetic fluvial topography to represent a 20 km × 40 km domain with a 160 m × 160 m grid spacing, as used in previous studies of glacial landscape evolution (Brædstrup et al., 2016; Magrani et al., 2021). Climate variables (e.g., air temperature, precipitation) were not explicitly defined in the glacier model. Instead, we used a temperature‐dependent mass balance function where accumulation and ablation are related to topographic height above or below the ELA, which is defined by the MAAT and the atmospheric lapse rate. We forced each simulation with change in MAAT (hereafter Δ*T*). The model was spun up to reach mass balance equilibrium with climate where Δ*T* at sea level was 7.0°C with a lapse rate of –6.0°C km^−1^. The resulting ice volume was used as the starting point for all further simulations, except where the simulation started with advance in which case the simulation was spun up to MAAT of 14°C.

The glacial erosion rate (*E*) was scaled by a non‐linear erosion law:

(1) $$E=K_{a}*{u_{b}}^{n}$$

where *u*_b_ is the velocity of basal sliding, *K*_a_ = 10^−6^ a m^−1^ is an empirical constant that is dependent on the hardness of the bedrock and the thermal properties of the glacier bed, and *n =* 2 is the sliding‐power coefficient (Herman et al., 2015; Koppes et al., 2015), to give transport‐limited conditions where sediment produced within the landscape was readily entrained by the glacier.

The glacier model deposited sediment at the bed by basal melting and entrained sediment that was either previously deposited or produced by glacial erosion from the bed. Sediment entrainment was assumed to be controlled by ice regelation into a granular bed. The rate of sediment entrainment, $v_{s}$, was implemented through Equation 29 in Egholm et al. (2012) and scaled with the thickness of englacial sediment, $h_{s}$, the effective pressure, N, and rate of basal melting, $m_{b}$:

(2) $$v_{s}=k_{g}*\frac{N}{h_{s}}-k_{s}*m_{b}$$

where $k_{g}$= 10^−7^ m^2^ Pa^−1^ a^−1^ and $k_{s}$= 0.1 are constants representing the apparent thermal conductivity of debris to ice (Alley et al., 1997; Iverson, 1993) and the basal ice debris concentration. Note that sediment entrainment occurred when the rate of ice regelation exceeded the rate of basal melting ( $v_{s}$ was positive) and sediment deposition occurred when melting outpaced regelation ( $v_{s}$ was negative). The relationship between effective pressure (i.e., the difference between the ice overburden pressure and subglacial water pressure) at the bed and regelation of sediment for a temperate glacier was determined experimentally by Iverson (1993), who showed that ice at the melting temperature regelated into a dense array of debris clasts and caused the clasts to move by entrainment. These experiments showed that the balance between sediment deposition by melting of basal ice and regelation of basal ice into a layer of debris clasts was driven by the ice temperature gradient that resulted from differences in effective pressure across the glacier bed (Iverson, 1993). Sediment collected from the bed was advected upwards, allowing the glacier to successively erode and entrain previously deposited moraines such that they might be partially or wholly erased. This model differs from that presented by Egholm et al. (2012) in that here sediment transport varied through the ice column in three dimensions rather than as one depth‐integrated layer, following the approach used in Rowan et al. (2015).

We tested the impact of climate change on glacier volume change and moraine building and preservation under a stable climate and a variable climate over timescales from 10^3^ to 10^4^ years. First, we imposed interannual variability to an otherwise stable climate forced by a random normal distribution of Δ*T* with a mean of 7°C and a standard deviation (*σ*_T_) of 0.5°C, 1.5°C or 3.0°C. In this experiment, the value for Δ*T* was constant for each year but varied randomly between years. The experiment was designed to be similar to that of Anderson et al. (2014) except that our simulations were not forced by a combined mass balance forcing including precipitation. Anderson et al. (2014) found that glaciers in Colorado experienced 10–15% length change due to interannual variability in climate; equivalent to *σ*_T_ of 1.5°C in our experiment. Second, we simulated advance and recession using step changes in Δ*T* between 7°C and 14°C (equivalent a change of 80% of the maximum glacier length) in increments of 0.35°C every 100 years for 2,000 years, and every 1,000 years for 10,000 years. Third, we imposed warming–cooling cycles over 2,000 years and 20,000 years using the same Δ*T* values as in previous simulations.

## 3 RESULTS

Moraines are identified in chronological order of formation (t_1−*n*_). Where the moraine numbering sequence is incomplete this indicates the removal of moraines formed earlier in the simulation. Changes in ice margin position are presented in terms of length change, where negative values indicate change of the trunk glacier margin in the up‐valley direction (recession) and positive values indicate change in the down‐valley direction (advance). We identified moraines from the sediment layer by their exceptional crest heights, and report changes in sediment‐covered area where the thickness exceeded the mean value at that point in time.

### 3.1 Moraine building without change in climate

In the spin‐up simulation, the e‐folding response time to reach mass balance equilibrium (i.e., when about two‐thirds of the change in glacier mass to ‘steady state’ was complete) with climate from an unglaciated domain was 188 years. The calculated response time was used to define the duration of the climatic forcing required for the glacier to respond completely to a change in climate. The glacier reached equilibrium then built a prominent moraine 224 m in relief close to the maximum ice extent over 500 years. The sediment‐covered area then gradually declined and the maximum moraine relief increased as subglacial sediment was moved to the ice margin.

**FIGURE 2:**
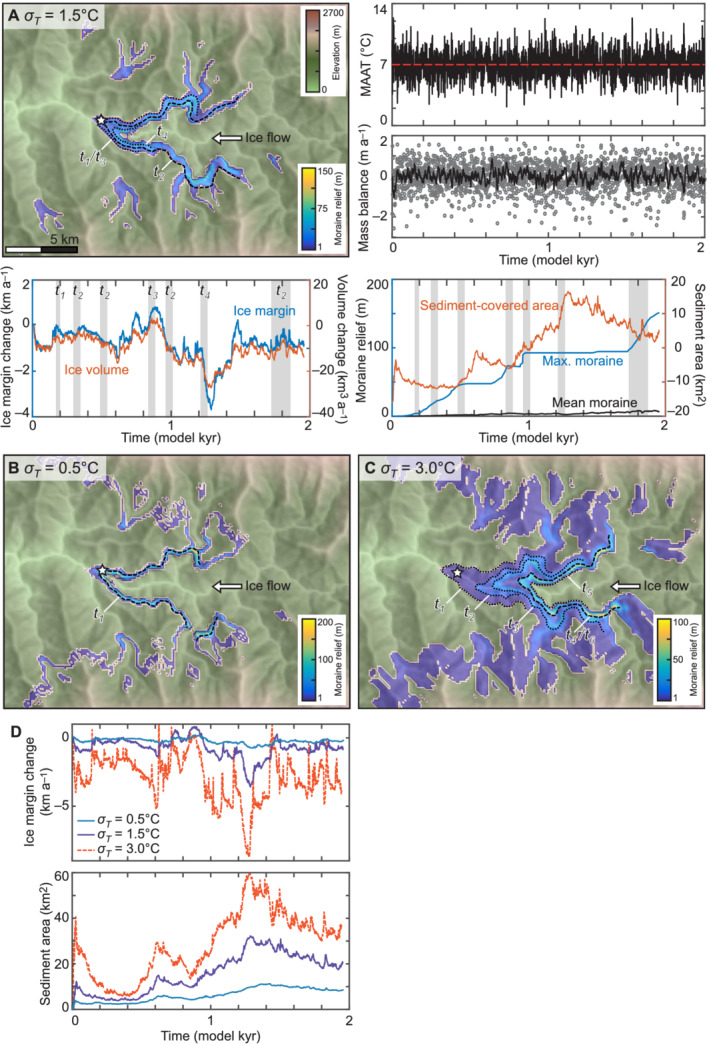
2,000‐year simulations with interannual variability in weather imposed on an otherwise stable climate. Moraine relief is shown for a standard deviation in mean annual air temperature (*σ* *T*) of (A) 1.5°C, (B) 0.5°C and (C) 3.0°C. the black lines in each map show the moraine location and their order of formation (t_*n*_), with the largest moraine shown by a dashed line and smaller moraines by dotted lines. The star indicates the position of the terminal moraine produced in the equilibrium simulation. Plots to the right of (A) show the climate forcing used as input to the simulation where *σ*_*T*_ = 1.5°C, and the resulting mass balance in metres water equivalent (w.e.) per year as annual values (grey points) with a 10‐year running mean (black line). Plots below (A) show fluctuations in the mean position of the ice margin along the main valley (i.e., glacier length change) and ice volume over time, and change in mean and maximum moraine relief and sediment‐covered area over time. Each plot is normalised relative to the start of the simulation where the ice margin is in mass balance equilibrium with the initial climate. Grey shading indicates the main stages of moraine building. Plots in (D) show the change in position of the ice margin and the change in sediment‐covered area for each simulation [Color figure can be viewed at wileyonlinelibrary.com]

With interannual variability in climate, as *σ*_T_ increased from 0.5°C to 3.0°C the number of moraines increased from one to five, the width of the area occupied by moraines increased, and the maximum moraine relief decreased from 213 m to 92 m (Figure 2). The ice margin excursion from the initial position that represented the glacier in mass balance equilibrium with climate increased from 1.2 km (0.4% of the maximum glacier length) to 9.6 km (34% of the maximum glacier length). In each case, the ice margin receded slightly within the first 200 years of the simulation because sediment accumulated at the initial maximum extent during the first 100 years (Figure 2D). The accumulation of sediment at the terminus caused the ice thickness here, and therefore the glacier extent, to reduce slightly. This effect is illustrated by the correlation in the timing of the increase in sediment‐covered area and the decrease in glacier extent, and the asymmetry in the direction of ice margin excursions (i.e., glacier length change). The ice margin fluctuated predominately in the up‐glacier direction (indicated by negative values for the ice margin excursion) and not equally on either side of the initial terminus position.

The smallest ice margin excursion from the initial position occurred where *σ*_T_ = 0.5°C to give −0.9 km and 0.3 km (a range of 1.2 km; 0.4% of the maximum glacier length) and one distinct moraine was built in the main valley (Figure 2B). When *σ*_T_ = 1.5°C, the ice margin excursion was −3.7 km to 0.8 km (4.5 km; 16% of the maximum glacier length) and formed three moraines (Figure 2A). The largest ice margin excursion occurred where *σ*_T_ = 3.0°C to give −8.7 km to 1.0 km (9.6 km; 34% of the maximum glacier length) and formed five moraines (Figure 2C). In the simulation where *σ*_T_ = 1.5°C, moraine t_1_ was built within 40 years. During the next 100 years, the glacier removed t_1_ and built a larger moraine t_2_ further up valley that gradually lengthened and increased in relief. Moraine t_2_ breached at both the terminus and where tributary glaciers met the trunk glacier at 950 years, and a smaller moraine t_3_ formed down valley from this position in the same location as t_1_. The glacier then receded slightly to rebuild t_2_ and form a smaller moraine t_4_ further up‐valley (Figure 2A).

### 3.2 Climate forcing shorter than the glacier response time

The 2,000‐year simulations were forced by step changes in Δ*T* at 100‐year intervals, chosen to be shorter than the glacier response time. Three simulations were made to represent a cooling climate (advance), a warming climate (recession), and warming–cooling cycles.

**FIGURE 3:**
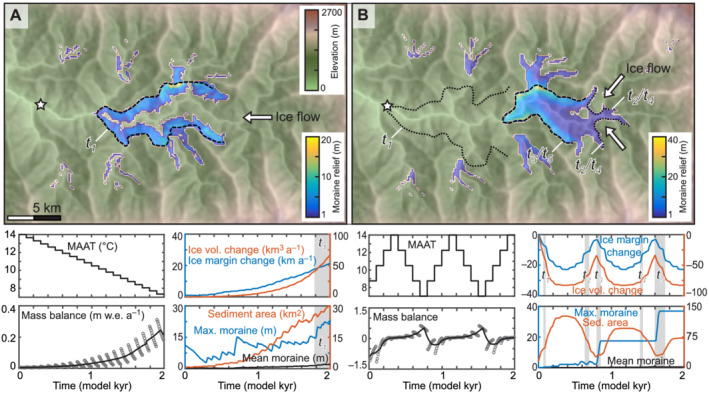
2,000‐year simulations with centennial changes in climate for (A) recession forced by warming of Δ*T* = 0.35°C at 100‐year intervals, and (B) symmetrical warming–cooling cycles. The symbology is the same as in Figure 2. Animated model results for these simulations are shown in the Supporting Information Videos [Link], [Link], [Link] [Color figure can be viewed at wileyonlinelibrary.com]

Under a cooling climate, cirque glaciers initiated and formed moraines 10–12 m in relief, then coalesced into a valley glacier that advanced 20 km from the initial cirque glaciers and entrained and redeposited these moraines down‐valley to form a single ridge. Small terminal moraines were preserved where cirque glaciers did not coalesce with the valley glacier and lateral moraines were partially preserved on hillslopes (Figure 3A and Supporting Information Video S1). A single terminal moraine t_1_ 20 m in relief and up to 1000 m wide occupied the main valley at the maximum extent of the glacier surrounded by lateral moraines of a similar relief and 200–400 m in width preserved on the hillslopes. Glacier volume expanded smoothly with cooling but the maximum moraine relief oscillated, with short‐lived erosion of the crest of 1 to 2 m every 100 years, to reach 23 m in relief at the end of the simulation (Figure 3A).

Under a warming climate, the glacier initially receded through the main valley, leaving a thin sheet of sediment a few metres thick that was reworked and had no identifiable moraine ridges. When the glacier reached the tributary valleys, a set of small but distinct moraines up to 7 m in relief formed with each Δ*T* (Supporting Information Video S3).

Under warming–cooling cycles, the mass balance response was asymmetrical, with rapid mass loss after the onset of warming and more gradual mass gain. Although the magnitude of Δ*T* was the same as for the single advance, the duration of cooling was not sufficient to reach mass balance equilibrium. Therefore, advances were less extensive, and the position of the highest‐relief moraine represented a substantially different length change to that of a single advance (Figure 3). At the onset of recession, a small moraine t_1_ was built at the maximum glacier extent and removed after 60 years (Figure 3B and Supporting Information Video S2). During the subsequent advance, moraine t_2_ started to form when the minimum Δ*T* was reached where the tributary glaciers coalesced at the valley floor and was removed in the following 100 years as the glacier continued to expand. Moraine t_3_ was built further down‐valley as the climate warmed (Figure 3B). After a further 100 years the glacier receded, then reoccupied t_3_ during the subsequent advance. During the first advance, the glacier took 50 years to build moraine t_3_ in the main valley with a maximum relief of 17 m. During the second period of advance, the height of t_3_ (now t_4_) increased to 36 m over 60 years. No erosion of the moraine took place as the glacier receded rapidly with the onset of warming. Although the magnitude of Δ*T* was the same here as in the simulation of a single advance, the duration of cooling was not sufficient for the glacier to reach equilibrium with climate, and therefore advances were less extensive and the position of the highest‐relief moraines represent substantially different glacier extents (Figure 3).

### 3.3 Climate forcing longer than the glacier response time

The 20,000‐year simulations were forced by step changes in Δ*T* at 1,000‐year intervals, chosen to be longer than the glacier response time.

Under a cooling climate, glacier volume change was similar to the shorter simulation, except that the glacier reached mass balance equilibrium with each Δ*T* and a greater volume of sediment was produced. A single narrow terminal moraine t_1_ 125 m in relief and 200–300 m wide occupied the main valley at the maximum extent surrounded by lateral moraines of a similar size. The maximum moraine continued to build as the glacier produced and transported sediment to the ice margins at the start of each cooling step (see Supporting Information Video S4).

**FIGURE 4:**
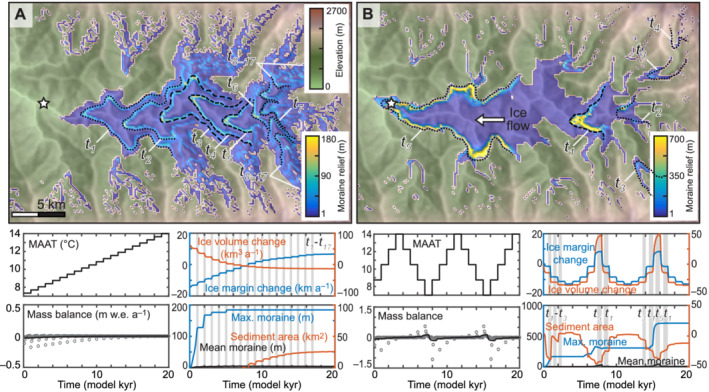
20,000‐year simulations with millennial changes in climate for (A) recession forced by warming of Δ*T* = 0.35°C at 1,000‐year intervals, and (B) symmetrical warming–cooling cycles. The symbology is the same as in Figure 2. Animated model results for these simulations are shown in the Supporting Information Videos [Link], [Link], [Link] [Color figure can be viewed at wileyonlinelibrary.com]

Under a warming climate, the glacier underwent a similar change in ice extent to the simulation with centennial climate forcing, but the net mass balance remained closer to zero as the glacier had sufficient time to reach mass balance equilibrium. A complete sequence of 17 moraines was preserved, with one moraine forming at the start of each Δ*T* until the cirque glaciers were too small to generate sufficient sediment. The moraine representing the maximum ice extent, t_5_, formed around 5 kyr and was 184 m in relief, with two preceding moraines (t_3_ and t_4_) and one subsequent moraine (t_6_) of a similar size (Figure 4A and Supporting Information Video S6).

Under warming–cooling cycles, two moraines greater than 100 m in relief were preserved in the main valley representing the major excursions during periods of cooling – one representing the maximum excursion of the glacier down‐valley (t_5_) and another representing the terminal position of a smaller valley glacier (t_1_) (Figure 4B and Supporting Information Video S5). Moraine t_1_ was substantially eroded during subsequent advances but not removed. Moraine t_5_ formed at the maximum extent of the first advance and was reoccupied and breached during the second advance. No further moraines were built in the main valley. Three smaller moraines (t_2_–t_4_) formed up‐valley from t_1_ during each recession and were reoccupied then removed during subsequent advances. Moraine t_1_ was the largest moraine and the maximum relief increased from 165 m at the start of the first warming period to 306 m during the second warming period, then to 710 m in the final warming period, with erosion of the moraine crest at the start of each advance of about 30 m over 1,000 years (Figure 4B).

## 4 DISCUSSION

### 4.1 Timescales of glacial erosion and sediment production

At the start of our simulations, sediment was produced as the glacier selectively eroded the fluvial landscape to a more glacial landscape, with erosion focussed around cirque accumulation areas and topographic highs in the main valley. The total volume of sediment produced was greater in the longer simulations and in simulations where the ice margin fluctuated more frequently than in simulations where glaciers only advanced (Figure 3). The formation of the highest‐relief moraines was more strongly influenced by the magnitude of ice margin excursions than the availability of sediment. Moraines with a similar relief formed under interannual variability in climate (i.e., due to variability in weather between years rather than climate change) to those moraines formed under climatic forcing of 20,000 years of advance or recession (92–213 m compared to 126 and 184 m), despite the total sediment yield being 76% lower (Figures 2 and 4). However, the larger interannual variability in the most extreme simulation (*σ*_T_ = 3.0°C) was equivalent to a change in glacier length of 34% (Figure 2D), which may be excessive in comparison with the observed magnitude of climatic variability during the Late Holocene. We consider the simulation where *σ*_T_ = 1.5°C to be representative of the response of a typical mountain glacier to interannual variability in climate as the magnitude of glacier length change was similar to that observed in Colorado by Anderson et al. (2014). We note that the distribution of ice margin excursions in our simulations was not symmetrical around the initial (climatic mean) margin position, as was the case in Anderson et al. (2014), because the accumulation of sediment at the glacier terminus limited the extent that the glacier could expand down‐valley (Figure 2D). Our results show that the feedback between sediment deposition and ice flow caused ice margin excursions in the up‐glacier direction to be 3–8 times greater than those in the down‐glacier direction.

As our experiments started from an unglaciated, fluvial landscape over timescales that were short relative to the timescales over which glacial erosion reshapes a landscape (Herman et al., 2018), simulated erosion focussed in the upper parts of the catchment rather than reshaping the valley floor more widely to create erosional feedbacks with glacier evolution (Anderson et al., 2012; Kaplan et al., 2009; MacGregor et al., 2009; Pedersen & Egholm, 2013). However, over Quaternary timescales, glacial erosion will dramatically reshape fluvial topography such that, where erosion outpaces rock uplift, moraines become more proximal through multiple glaciations because the hypsometry of the landscape influences glacier mass balance (Anderson et al., 2012; Brocklehurst & Whipple, 2004; Pedersen & Egholm, 2013; Whipple et al., 1999). Based on previous studies of Quaternary glacial landscape evolution, we expect the extent of advances and therefore the position of terminal moraines to become more proximal over successive glaciations when the rate of glacial erosion outpaces the rate of rock uplift – the ‘far‐flung moraines’ hypothesis (Anderson et al., 2012). As Anderson et al. (2012) demonstrate, this process favours the preservation of older moraines located beyond the limits of later glacial erosion. Previous glacier modelling has shown that the extent of glacier expansion in response to climatic forcing changes with the number of glaciations, from near‐linear in an unglaciated fluvial landscape to highly non‐linear in a glacially eroded landscape when the ELA (snowline) reaches the hypsometric maximum (Pedersen & Egholm, 2013). The tendency for glacier expansion to reduce in magnitude with ongoing erosion can be overcome by a change in climatic forcing such as the mid‐Pleistocene transition around 1 Ma, which resulted in shorter‐lived, but more expansive, glaciations in a glacially preconditioned landscape compared to in an unglaciated landscape (Pedersen & Egholm, 2013). We therefore expect that moraines formed when glaciation occurs across an initially unglaciated landscape (as is the case in our simulations) will provide the most straightforward record of palaeoclimate change, and the preservation potential of these moraines over Quaternary timescales will be good unless the rate of rock uplift exceeds the rate of glacial erosion.

Moraine formation in landscapes that were previously glaciated occurs more frequently and is less straightforward to interpret. Some of the oldest mountain glacier moraines identified using terrestrial cosmogenic nuclide exposure‐age dating are older than the mid‐Pleistocene transition (e.g., Hein et al., 2011; Smith et al., 2005) and could be used to explore variability in the extent of glaciations in response to landscape modification through the Quaternary, but this is beyond the scope of our study. Over Late Quaternary timescales, the direction of change in climate will also affect moraine preservation regardless of topographic feedbacks, as if the climate becomes less conducive to glacier expansion then moraine preservation becomes more likely. For example, older glacial landforms are more frequent in the arid rather than monsoon‐influenced Himalaya where increasing monsoon intensity promoted extensive Late Glacial and Holocene glaciation and enhanced fluvial and hillslope erosion that partially or completely removed older moraines (Owen et al., 2005).

The erosion rate was calibrated such that the glacier was readily able to entrain new sediment as it was produced. If the erosion rate was lower, then the relative moraine relief would reduce but the patterns of moraine formation would be similar, because moraine relief is controlled by the magnitude of the ice margin fluctuations. If the erosion rate was higher, such that large sediment volumes accumulated at the glacier bed, this would not necessarily lead to an increase in ice flow (Zoet & Iverson, 2020) and therefore the resulting moraine sequences would be similar to those formed by a glacier flowing over a rock bed where sediment flux was lower. In tectonically active mountain ranges, high rates of glacial erosion can produce more sediment than can be exported to form moraines when the net annual glacier mass balance is close to zero or negative (Scherler et al., 2011). Sediment accumulates supraglacially and insulates the ice surface, resulting in a decoupling of the glacier mass balance from climatic forcing and the potential for the glacier to expand without any change in climate (Anderson et al., 2018; Rowan et al., 2015). Debris‐covered glaciers can form ice‐cored moraines that are abandoned and then reactivated during a later advance, resulting in composite landforms with a complex geochronology (Crump et al., 2017; Kirkbride & Brazier, 1998). Therefore, moraines formed by debris‐covered glaciers can be more challenging to interpret in the context of palaeoclimate change compared to those formed by relatively clean‐ice glaciers over a similar timescale to the glacier response time, such as during the Late Holocene (Solomina et al., 2016). However, while palimpsest landforms may be difficult to identify in the field, surface exposure‐age dating can identify distinct periods of moraine formation (e.g., Peltier et al., 2021). When interpreting changes in palaeoclimate from moraines over longer (Quaternary) timescales, debris‐covered glaciers are less likely to be a concern, because large changes in ice volume will result in glaciers exporting most supraglacial debris to the ice margins at the start of each advance before moraine building is complete (Rowan et al., 2015).

### 4.2 Controls on moraine formation

Moraine formation is not instantaneous, even over the geological timescales of Quaternary climate change (Crump et al., 2017; Kirkbride & Winkler, 2012). Our results support the observation of Anderson et al. (2014) that moraines tens of metres in relief can be built in about 50 years. However, we also demonstrate that moraine building can be a discontinuous process, with individual moraines being partially eroded when the glacier recedes before the moraine crests are reconstructed during subsequent advances if the glacier expands to the same position (Figure 3B). In this case, the timescale for building a single moraine can be several thousands of years, with shorter (10^1^–10^2^ years) periods of sediment deposition interspersed with longer (10^2^–10^3^ years) periods of no sediment accumulation and some erosion of the landform; as mentioned earlier, surface exposure‐age dating can separate these events and may help to explain the sometimes wide distribution of exposure ages of individual moraines as a geomorphological rather than geochronological source of uncertainty (Kirkbride & Winkler, 2012). A series of additions to a single moraine are likely to result in a distribution of exposure‐ages that vary both across the moraine and with depth (Tomkins et al., 2021).

An important control on the geometry of moraines is the timescale over which the ice margin fluctuates, as, given more time, a glacier will consolidate pre‐existing deposits. The 20,000‐year recession simulation produced an order of magnitude more sediment than the 2,000‐year recession, and this sediment was distributed into moraines that occupied only one‐third of the area of those produced in the shorter simulation (Figure 4). If an ice margin remains relatively stable over timescales of centuries rather than decades, entrainment and redeposition of subglacial sediment will result in narrower ice‐marginal moraines than those formed during a shorter time period (cf. Figures 3 and 4), similar to the moraines formed by the southern Laurentide Ice Sheet (Clark, 1992).

The duration, rather than the magnitude, of change in climate relative to the glacier response time is important for the timing of moraine formation. Under warming–cooling cycles where the rate of Δ*T* was greater than the response time, moraine formation was contemporary to the occurrence of maximum climate cooling (Figure 4B). However, when the rate of Δ*T* was shorter than the glacier response time, moraine building began with the onset of recession and was completed 50 years after the end of the maximum cooling (Figure 3B). The duration of change in climate relative to the glacier response time is also important for the position of moraine formation. In the 20,000‐year simulation, the glacier was able to advance twice as far down‐valley than in the 2,000‐year simulation, as the duration of changes in Δ*T* were greater than the response time (Figure 3A). Only the 20,000‐year recession generated a complete set of moraines (t_1_–t_17_) representing each Δ*T* as the glacier margin stabilised with each Δ*T* (Figure 4A). This sequence of landforms is similar to the postglacial moraines observed in the central Southern Alps, New Zealand, that formed during a period of overall recession (Denton et al., 2021).

Hypsometry is a primary control on glacier extent, as when the ELA reached the low‐relief topography in the main valley, the rate of glacier length change with Δ*T* increased compared to in the steeper tributary valleys, because a small change in ELA represents a larger area of the valley. Therefore, moraine spacing in the main valley was greater than in the tributary valleys despite no difference in the amount of palaeoclimate change (i.e., the magnitude of Δ*T* was the same). The largest moraines often formed at two pinning points: (1) the break in slope where tributary glaciers coalesced at the head of the main valley; (2) at a bend in the middle of the main valley (Figures 3B and 4). These patterns are observed in glacial landscapes where topography promotes ice margin stability, for example, where a break in slope occurs, as seen in north‐eastern Russia (Barr & Clark, 2012) and where valley width changes, as seen in southern Norway (Lukas, 2007); more detailed discussions are presented in Barr & Lovell (2014) and Mackintosh et al. (2017).

### 4.3 Controls on moraine preservation

The most distal Holocene moraines are not necessarily the oldest. In the 20,000‐year simulations, the moraines with the highest relief formed where the tributary glaciers coalesced in the upper main valley. However, these moraines represented different phases of the sequence of glacier change; during recession this position was occupied by moraine t_5_ (Δ*T* = 9.1°C) whereas under warming–cooling cycles, this position was occupied by moraine t_1_ (Δ*T* = 8.75°C), which had nearly four times greater relief (Figure 4) suggesting a greater likelihood of preservation of the moraine formed under a fluctuating climate rather than a warming climate. Moreover, the large t_1_ moraine limited the expansion of subsequent advances, creating a non‐linear response in glacier length change with climatic forcing and enhancing the likelihood of moraine preservation by preventing ice from overriding, eroding, and re‐entraining the sediment. The crest of such a moraine is likely to represent the exposure age of the landform as contemporary to the initial formation of the moraine in response to a single phase of ice volume change more accurately than a moraine that as undergone episodes of erosion and addition of sediment. The other moraines formed during the first warming–cooling cycle were progressively eroded as the glacier reoccupied these positions during subsequent warming–cooling cycles (Figure 3B and 4B). Therefore, surface‐exposure ages from moraine sequences formed during recession are likely to be more consistent across each landform and give more accurate ages for moraine formation, assuming that the sedimentology, and so landform stability, are similar (Tomkins et al., 2021). Recessional moraine sequences, such as those formed after Termination 1 by the Pukaki and Rakaia glaciers in the central Southern Alps, New Zealand, are also most likely to provide a complete record of palaeoclimate change (Figure 4A) (e.g., Denton et al., 2021; Putnam et al., 2013).

Through simulations of multiple warming–cooling cycles, more distal moraines were less likely to be preserved and were in some cases removed (Figure 3B), giving the impression of more modest glacier length change, and therefore palaeoclimate cooling, than was the case. However, over Quaternary timescales, more distal moraines may represent much older glaciations, as when glacial erosion exceeds the rate of rock uplift, glaciers can erode the valley floors sufficiently to lower the ELA and reduce the extent of later advances (Anderson et al., 2012; MacGregor et al., 2009). In two simulations, terminal moraines were breached by later glacier expansion. Although not included in our simulations, fluvial erosion will preferentially remove terminal moraines that cross valley floors rather than lateral moraines occupying the hillslopes such that lateral moraines may be better preserved, particularly in arid climates where mass movement is less frequent (Fu & Yi, 2009).

In comparison with mountain glaciers that respond rapidly to small variations in climate, ice sheets have long response times and are therefore coarser filters of climate signals (Bahr et al., 1998; Jóhannesson et al., 1989). The moraine sequences formed by ice sheets may contain more gaps and are more likely to reflect only larger palaeoclimate excursions, giving the misleading impression of relatively slow climate change (Hughes et al., 2016). Our results imply that mountain glaciers with their relatively short response times can build more complete sequences of moraines than larger ice masses. However, the moraine sequences formed during shorter warming–cooling cycles may be relatively poorly preserved, as these smaller moraines are more readily overrun and eroded by subsequent glaciations. While moraines produced over longer timescales are broadly more likely to be preserved both because the volume of sediment generated by erosion and available to form moraines is greater and because longer‐term ice margin stability allows sediment to be consolidated into narrower, higher‐relief moraines, the largest moraine may not represent the maximum ice extent. The largest landforms present more formidable barriers to an advancing glacier than smaller moraines and can restrict subsequent advances to similar limits, thus recording a smaller‐than‐expected change in glacier extent while promoting the preservation of more distal moraines.

## 5 CONCLUSIONS

Ice‐marginal moraine sequences can be diagnostic of terrestrial palaeoclimate conditions, however, sediment availability and the relationship between the duration of palaeoclimate change and the glacier response time will affect the geometry and geochronology of moraine building and moraine preservation. Longer warming–cooling cycles (10^4^ years) produced larger, narrower moraines than shorter warming–cooling cycles (10^3^ years) with the same magnitude of change in Δ*T*. An advancing glacier will record the maximum expansion in ice volume by building a terminal moraine, and the size of this moraine reflects the amount of sediment available and therefore the erosional history of the glacier. Recessional moraines representing a complete record of deglaciation can be preserved if the glacier response time is shorter than the rate of change in climate sufficient to force the ice margin to migrate; in this case, Δ*T* of 0.35°C per 100 years compared to the e‐folding response time to reach mass balance equilibrium of 188 years.

We demonstrate that the moraines resulting from both transient and equilibrium glacier volume changes can be preserved over timescales representative of Late Quaternary palaeoclimate change. For glaciers capable of eroding and transporting sufficient volumes of sediment and building prominent moraines, the response time is likely to exceed the moraine building time, and therefore both transient and equilibrium changes in ice volume are recorded by moraine formation. However, our results also show that glaciers can form three high‐relief moraines even in the absence of a change in climate due to ice margin fluctuations forced by interannual variability in climate (i.e., variations in weather between years). Therefore, interpretations of glacial geomorphology as a palaeoclimate archive should consider the geometry and positions of individual moraines as indicators of the duration of ice margin stability and in context of the glacier's dynamic response time.

## Supporting information


**Video S1.**2 kyr advance

**Video S2.**2 kyr cycles

**Video S3.**2 kyr recession

**Video S4.**20 kyr advance

**Video S5.**20 kyr cycles

**Video S6.**20 kyr recession

## Data Availability

No new data were created in this study. Animations of glacier model output for each experiment are included as Supporting Information for those where a change in climate was imposed. Animations of the outputs of the interannual variability simulations are available from Zenodo (10.5281/zenodo.6337406).

## References

[esp5371-bib-0001] Alley, R.B. , Cuffey, K.M. , Evenson, E.B. , Strasser, J.C. , Lawson, D.E. & Larson, G.J. (1997) How glaciers entrain and transport basal sediment: Physical constraints. Quaternary Science Reviews, 16(9), 1017–1038. Available from: 10.1016/S0277-3791(97)00034-6

[esp5371-bib-0002] Anderson, L.S. , Geirsdóttir, Á. , Flowers, G.E. , Wickert, A.D. , Aðalgeirsdóttir, G. & Thorsteinsson, T. (2019) Controls on the lifespans of Icelandic ice caps. Earth and Planetary Science Letters, 527, 115780. Available from: 10.1016/j.epsl.2019.115780

[esp5371-bib-0003] Anderson, L.S. , Roe, G.H. & Anderson, R.S. (2014) The effects of interannual climate variability on the moraine record. Geology, 42(1), 55–58. Available from: 10.1130/G34791.1

[esp5371-bib-0004] Anderson, R.S. , Anderson, L.S. , Armstrong, W.H. , Rossi, M.W. & Crump, S.E. (2018) Glaciation of alpine valleys: The glacier – debris‐covered glacier – rock glacier continuum. Geomorphology, 311, 127–142. Available from: 10.1016/j.geomorph.2018.03.015

[esp5371-bib-0005] Anderson, R.S. , Dühnforth, M. , Colgan, W. & Anderson, L. (2012) Far‐flung moraines: Exploring the feedback of glacial erosion on the evolution of glacier length. Geomorphology, 179, 269–285. Available from: 10.1016/j.geomorph.2012.08.018

[esp5371-bib-0006] Bahr, D.B. , Pfeffer, W.T. , Sassolas, C. & Meier, M.F. (1998) Response time of glaciers as a function of size and mass balance: 1. Theory. Journal of Geophysical Research: Solid Earth, 103(B5), 9777–9782. Available from: 10.1029/98JB00507

[esp5371-bib-0007] Bakke, J. , Paasche, Ø. , Schaefer, J.M. & Timmermann, A. (2021) Long‐term demise of sub‐Antarctic glaciers modulated by the Southern Hemisphere Westerlies. Scientific Reports, 11(1), 8361. Available from: 10.1038/s41598-021-87317-5 33863941 PMC8052370

[esp5371-bib-0008] Balco, G. (2020) Glacier Change and Paleoclimate Applications of Cosmogenic‐Nuclide Exposure Dating. Annual Review of Earth and Planetary Sciences, 48(1), 21–48. Available from: 10.1146/annurev-earth-081619-052609

[esp5371-bib-0009] Barr, I.D. & Clark, C.D. (2012) Late Quaternary glaciations in Far NE Russia; combining moraines, topography and chronology to assess regional and global glaciation synchrony. Quaternary Science Reviews, 53, 72–87. Available from: 10.1016/j.quascirev.2012.08.004

[esp5371-bib-0010] Barr, I.D. & Lovell, H. (2014) A review of topographic controls on moraine distribution. Geomorphology, 226, 44–64. Available from: 10.1016/j.geomorph.2014.07.030

[esp5371-bib-0011] Boston, C.M. , Lukas, S. & Carr, S.J. (2015) A Younger Dryas plateau icefield in the Monadhliath, Scotland, and implications for regional palaeoclimate. Quaternary Science Reviews, 108, 139–162. Available from: 10.1016/j.quascirev.2014.11.020

[esp5371-bib-0012] Brædstrup, C.F. , Egholm, D.L. , Ugelvig, S.V. & Pedersen, V.K. (2016) Basal shear stress under alpine glaciers: insights from experiments using the iSOSIA and Elmer/Ice models. Earth Surface Dynamics, 4(1), 159–174. Available from: 10.5194/esurf-4-159-2016

[esp5371-bib-0013] Brocklehurst, S.H. & Whipple, K.X. (2004) Hypsometry of glaciated landscapes. Earth Surface Processes and Landforms, 29(7), 907–926. Available from: 10.1002/esp.1083

[esp5371-bib-0014] Broecker, W.S. (2003) Does the Trigger for Abrupt Climate Change Reside in the Ocean or in the Atmosphere? Science, 300(5625), 1519–1522. Available from: 10.1126/science.1083797 12791974

[esp5371-bib-0015] Burke, E.E. & Roe, G.H. (2014) The absence of memory in the climatic forcing of glaciers. Climate Dynamics, 42(5‐6), 1335–1346. Available from: 10.1007/s00382-013-1758-0

[esp5371-bib-0016] Chandler, B.M.P. , Chandler, S.J.P. , Evans, D.J.A. , Ewertowski, M.W. , Lovell, H. , Roberts, D.H. et al. (2020) Sub‐annual moraine formation at an active temperate Icelandic glacier. Earth Surface Processes and Landforms, 45(7), 1622–1643. Available from: 10.1002/esp.4835

[esp5371-bib-0017] Chandler, B.M.P. , Evans, D.J.A. & Roberts, D.H. (2016) Characteristics of recessional moraines at a temperate glacier in SE Iceland: Insights into patterns, rates and drivers of glacier retreat. Quaternary Science Reviews, 135, 171–205. Available from: 10.1016/j.quascirev.2016.01.025

[esp5371-bib-0018] Clark, p.U. (1992) Surface form of the southern Laurentide Ice Sheet and its implications to ice‐sheet dynamics. Geological Society of America Bulletin, 11(5), 595–605. Available from: 10.1130/0016-7606(1992)104<0595:SFOTSL>2.3.CO;2

[esp5371-bib-0019] Clark, P.U. , Shakun, J.D. , Baker, P.A. , Bartlein, P.J. , Brewer, S. , Brook, E. et al. (2012) Global climate evolution during the last deglaciation. Proceedings of the National Academy of Sciences, 109(19), E1134–E1142. Available from: 10.1073/pnas.1116619109 PMC3358890 22331892

[esp5371-bib-0020] Crump, S.E. , Anderson, L.S. , Miller, G.H. & Anderson, R.S. (2017) Interpreting exposure ages from ice‐cored moraines: a Neoglacial case study on Baffin Island, Arctic Canada. Journal of Quaternary Science, 32(8), 1049–1062. Available from: 10.1002/jqs.2979

[esp5371-bib-0021] Denton, G.H. , Anderson, R.F. , Toggweiler, J.R. , Edwards, R.L. , Schaefer, J.M. & Putnam, A.E. (2010) The Last Glacial Termination. Science, 328(5986), 1652–1656. Available from: 10.1126/science.1184119 20576882

[esp5371-bib-0022] Denton, G.H. , Putnam, A.E. , Russell, J.L. , Barrell, D.J.A. , Schaefer, J.M. , Kaplan, M.R. & Strand, p.D. (2021) The Zealandia Switch: Ice age climate shifts viewed from Southern Hemisphere moraines. Quaternary Science Reviews, 257, 106771. Available from: 10.1016/j.quascirev.2020.106771

[esp5371-bib-0023] Doughty, A.M. , Mackintosh, A.N. , Anderson, B.M. , Dadic, R. , Putnam, A.E. , Barrell, D.J.A. et al. (2017) An exercise in glacier length modeling: Interannual climatic variability alone cannot explain Holocene glacier fluctuations in New Zealand. Earth and Planetary Science Letters, 470, 48–53. Available from: 10.1016/j.epsl.2017.04.032

[esp5371-bib-0024] Egholm, D.L. , Knudsen, M.F. , Clark, C.D. & Lesemann, J.E. (2011) Modeling the flow of glaciers in steep terrains: The integrated second‐order shallow ice approximation (iSOSIA). Journal of Geophysical Research: Earth Surface, 116(F2). Available from: 10.1029/2010JF001900

[esp5371-bib-0025] Egholm, D.L. , Pedersen, V.K. , Knudsen, M.F. & Larsen, N.K. (2012) Coupling the flow of ice, water, and sediment in a glacial landscape evolution model. Geomorphology, 141–142, 47–66. Available from: 10.1016/j.geomorph.2011.12.019

[esp5371-bib-0026] Ely, J.C. , Clark, C.D. , Hindmarsh, R.C.A. , Hughes, A.L.C. , Greenwood, S.L. , Bradley, S.L. , et al. (2021) Recent progress on combining geomorphological and geochronological data with ice sheet modelling, demonstrated using the last British–Irish Ice Sheet. Journal of Quaternary Science, 36(5), 946–960. Available from: 10.1002/jqs.3098

[esp5371-bib-0027] Fu, P. & Yi, C. (2009) Relationships between the heights of moraines and lengths of former glaciers in Tibet and surrounding mountains. Geomorphology, 103(2), 205–211. Available from: 10.1016/j.geomorph.2008.04.023

[esp5371-bib-0028] Gibbons, A.B. , Megeath, J.D. & Pierce, K.L. (1984) Probability of moraine survival in a succession of glacial advances. Geology, 12(6), 327–330. Available from: 10.1130/0091-7613(1984)12<327:POMSIA>2.0.CO;2

[esp5371-bib-0029] Greenwood, S.L. & Clark, C.D. (2009) Reconstructing the last Irish Ice Sheet 1: changing flow geometries and ice flow dynamics deciphered from the glacial landform record. Quaternary Science Reviews, 28(27‐28), 3085–3100. Available from: 10.1016/j.quascirev.2009.09.008

[esp5371-bib-0030] Haeberli, W. (1995) Glacier fluctuations and climate change detection. Geografia Fisica e Dinamica Quaternaria, 18, 191–199.

[esp5371-bib-0031] Hein, A.S. , Dunai, T.J. , Hulton, N.R.J. & Xu, S. (2011) Exposure dating outwash gravels to determine the age of the greatest Patagonian glaciations. Geology, 39(2), 103–106. Available from: 10.1130/G31215.1

[esp5371-bib-0032] Herman, F. , Beyssac, O. , Brughelli, M. , Lane, S.N. , Leprince, S. , Adatte, T. et al. (2015) Erosion by an Alpine glacier. Science, 350(6257), 193–195. Available from: 10.1126/science.aab2386 26450208

[esp5371-bib-0033] Herman, F. , Braun, J. , Deal, E. & Prasicek, G. (2018) The Response Time of Glacial Erosion. Journal of Geophysical Research: Earth Surface, 123(4), 801–817. Available from: 10.1002/2017JF004586 PMC6055901 30069424

[esp5371-bib-0034] Hughes, A.L.C. , Gyllencreutz, R. , Lohne, Ø.S. , Mangerud, J. & Svendsen, J.I. (2016) The last Eurasian ice sheets – a chronological database and time‐slice reconstruction, DATED‐1. Boreas, 45(1), 1–45. Available from: 10.1111/bor.12142

[esp5371-bib-0035] Huybers, K. & Roe, G.H. (2009) Spatial Patterns of Glaciers in Response to Spatial Patterns in Regional Climate. Journal of Climate, 22(17), 4606–4620. Available from: 10.1175/2009JCLI2857.1

[esp5371-bib-0075] Iverson, N.R. (1993) Regelation of ice through debris at glacier beds: Implications for sediment transport. Geology, 21(6), 559–562.

[esp5371-bib-0036] Jóhannesson, T. , Raymond, C. & Waddington, E.D. (1989) Timescale for adjustment of glaciers to changes in mass balance. Journal of Glaciology, 35(121), 355–369. Available from: 10.1017/S002214300000928X

[esp5371-bib-0037] Kaplan, M.R. , Hein, A.S. , Hubbard, A. & Lax, S.M. (2009) Can glacial erosion limit the extent of glaciation? Geomorphology, 103(2), 172–179. Available from: 10.1016/j.geomorph.2008.04.020

[esp5371-bib-0038] Kaspari, S. , Hooke, R.L.B. , Mayewski, P.A. , Kang, S. , Hou, S. & Qin, D. (2008) Snow accumulation rate on Qomolangma (Mount Everest), Himalaya: synchroneity with sites across the Tibetan Plateau on 50–100 year timescales. Journal of Glaciology, 54(185), 343–352. Available from: 10.3189/002214308784886126

[esp5371-bib-0039] Kaufman, D. , McKay, N. , Routson, C. , Erb, M. , Davis, B. , Heiri, O. et al. (2020) A global database of Holocene paleotemperature records. Scientific Data, 7(1), 115. Available from: 10.1038/s41597-020-0445-3 32286335 PMC7156486

[esp5371-bib-0040] Kelly, M.A. , Russell, J.M. , Baber, M.B. , Howley, J.A. , Loomis, S.E. , Zimmerman, S. et al. (2014) Expanded glaciers during a dry and cold Last Glacial Maximum in equatorial East Africa. Geology, 42(6), 519–522. Available from: 10.1130/G35421.1

[esp5371-bib-0041] Kirkbride, M.P. & Brazier, V. (1998) A critical evaluation of the use of glacier chronologies in climatic reconstruction, with reference to New Zealand. Quaternary Proceedings, 6, 55–64.

[esp5371-bib-0042] Kirkbride, M.P. & Winkler, S. (2012) Correlation of Late Quaternary moraines: impact of climate variability, glacier response, and chronological resolution. Quaternary Science Reviews, 46, 1–29. Available from: 10.1016/j.quascirev.2012.04.002

[esp5371-bib-0043] Koppes, M. , Hallet, B. , Rignot, E. , Mouginot, J. , Wellner, J.S. & Boldt, K. (2015) Observed latitudinal variations in erosion as a function of glacier dynamics. Nature, 526(7571), 100–103. Available from: 10.1038/nature15385 26432248

[esp5371-bib-0044] Lisiecki, L.E. & Raymo, M.E. (2005) A Pliocene‐Pleistocene stack of 57 globally distributed benthic δ ^18^ O records. Paleoceanography, 20(1), Available from: 10.1029/2004PA001071

[esp5371-bib-0045] Lukas, S. (2007) Early‐Holocene glacier fluctuations in Krundalen, south central Norway: palaeoglacier dynamics and palaeoclimate. The Holocene, 17(5), 585–598. Available from: 10.1177/0959683607078983

[esp5371-bib-0046] Lukas, S. , Graf, A. , Coray, S. & Schlüchter, C. (2012) Genesis, stability and preservation potential of large lateral moraines of Alpine valley glaciers – towards a unifying theory based on Findelengletscher, Switzerland. Quaternary Science Reviews, 38, 27–48. Available from: 10.1016/j.quascirev.2012.01.022

[esp5371-bib-0047] MacGregor, K.R. , Anderson, R.S. & Waddington, E.D. (2009) Numerical modeling of glacial erosion and headwall processes in alpine valleys. Geomorphology, 103(2), 189–204. Available from: 10.1016/j.geomorph.2008.04.022

[esp5371-bib-0048] Mackintosh, A.N. , Anderson, B.M. & Pierrehumbert, R.T. (2017) Reconstructing Climate from Glaciers. Annual Review of Earth and Planetary Sciences, 45(1), 649–680. Available from: 10.1146/annurev-earth-063016-020643

[esp5371-bib-0049] Magrani, F. , Valla, P.G. & Egholm, D. (2021) Modelling alpine glacier geometry and subglacial erosion patterns in response to contrasting climatic forcing. Earth Surface Processes and Landforms, esp.5302(4), 1054–1072. Available from: 10.1002/esp.5302

[esp5371-bib-0050] Menounos, B. , Goehring, B.M. , Osborn, G. , Margold, M. , Ward, B. , Bond, J. et al. (2017) Cordilleran Ice Sheet mass loss preceded climate reversals near the Pleistocene Termination. Science, 358(6364), 781–784. Available from: 10.1126/science.aan3001 29123066

[esp5371-bib-0051] Muzikar, P. (2016) Explicit solutions for a probabilistic moraine preservation model. Journal of Glaciology, 62(236), 1181–1185. Available from: 10.1017/jog.2016.109

[esp5371-bib-0052] Overland, J.E. , Dethloff, K. , Francis, J.A. , Hall, R.J. , Hanna, E. , Kim, S.‐J. et al. (2016) Nonlinear response of mid‐latitude weather to the changing Arctic. Nature Climate Change, 6(11), 992–999. Available from: 10.1038/nclimate3121

[esp5371-bib-0053] Owen, L.A. , Finkel, R.C. , Barnard, P.L. , Haizhou, M. , Asahi, K. , Caffee, M.W. & Derbyshire, E. (2005) Climatic and topographic controls on the style and timing of Late Quaternary glaciation throughout Tibet and the Himalaya defined by 10Be cosmogenic radionuclide surface exposure dating. Quaternary Science Reviews, 24(12‐13), 1391–1411. Available from: 10.1016/j.quascirev.2004.10.014

[esp5371-bib-0054] Pedersen, V.K. & Egholm, D.L. (2013) Glaciations in response to climate variations preconditioned by evolving topography. Nature, 493(7431), 206–210. Available from: 10.1038/nature11786 23302860

[esp5371-bib-0055] Peltier, C. , Kaplan, M.R. , Birkel, S.D. , Soteres, R.L. , Sagredo, E.A. , Aravena, J.C. et al. (2021) The large MIS 4 and long MIS 2 glacier maxima on the southern tip of South America. Quaternary Science Reviews, 262, 106858. Available from: 10.1016/j.quascirev.2021.106858

[esp5371-bib-0074] Penck, A. (1905) Glacial features in the surface of the Alps. The Journal of Geology, 13(1), 1–19.

[esp5371-bib-0056] Putnam, A.E. , Schaefer, J.M. , Denton, G.H. , Barrell, D.J.A. , Birkel, S.D. , Andersen, B.G. et al. (2013) The Last Glacial Maximum at 44°S documented by a 10Be moraine chronology at Lake Ohau, Southern Alps of New Zealand. Quaternary Science Reviews, 62, 114–141. Available from: 10.1016/j.quascirev.2012.10.034

[esp5371-bib-0057] Roe, G.H. & O'Neal, M.A. (2009) The response of glaciers to intrinsic climate variability: observations and models of late‐Holocene variations in the Pacific Northwest. Journal of Glaciology, 55(193), 839–854. Available from: 10.3189/002214309790152438

[esp5371-bib-0058] Rowan, A.V. , Egholm, D.L. , Quincey, D.J. & Glasser, N.F. (2015) Modelling the feedbacks between mass balance, ice flow and debris transport to predict the response to climate change of debris‐covered glaciers in the Himalaya. Earth and Planetary Science Letters, 430, 427–438. Available from: 10.1016/j.epsl.2015.09.004

[esp5371-bib-0059] Scherler, D. , Bookhagen, B. & Strecker, M.R. (2011) Hillslope‐glacier coupling: The interplay of topography and glacial dynamics in High Asia. Journal of Geophysical Research: Earth Surface 116, 116(F2), Available from: 10.1029/2010JF001751

[esp5371-bib-0060] Seguinot, J. , Ivy‐Ochs, S. , Jouvet, G. , Huss, M. , Funk, M. & Preusser, F. (2018) Modelling last glacial cycle ice dynamics in the Alps. The Cryosphere, 12(10), 3265–3285. Available from: 10.5194/tc-12-3265-2018

[esp5371-bib-0061] Shakun, J.D. , Clark, P.U. , He, F. , Marcott, S.A. , Mix, A.C. , Liu, Z. et al. (2012) Global warming preceded by increasing carbon dioxide concentrations during the last deglaciation. Nature, 484(7392), 49–54. Available from: 10.1038/nature10915 22481357

[esp5371-bib-0062] Sime, L.C. , Wolff, E.W. , Oliver, K.I.C. & Tindall, J.C. (2009) Evidence for warmer interglacials in East Antarctic ice cores. Nature, 462(7271), 342–345. Available from: 10.1038/nature08564 19924212

[esp5371-bib-0063] Smedley, R.K. , Chiverrell, R.C. , Ballantyne, C.K. , Burke, M.J. , Clark, C.D. , Duller, G.A.T. et al. (2017) Internal dynamics condition centennial‐scale oscillations in marine‐based ice‐stream retreat. Geology, 45(9), 787–790. Available from: 10.1130/G38991.1

[esp5371-bib-0064] Smith, J.A. , Finkel, R.C. , Farber, D.L. , Rodbell, D.T. & Seltzer, G.O. (2005) Moraine preservation and boulder erosion in the tropical Andes: interpreting old surface exposure ages in glaciated valleys. Journal of Quaternary Science, 20(7‐8), 735–758. Available from: 10.1002/jqs.981

[esp5371-bib-0065] Solomina, O.N. , Bradley, R.S. , Hodgson, D.A. , Ivy‐Ochs, S. , Jomelli, V. , Mackintosh, A.N. et al. (2015) Holocene glacier fluctuations. Quaternary Science Reviews, 111, 9–34. Available from: 10.1016/j.quascirev.2014.11.018

[esp5371-bib-0066] Solomina, O.N. , Bradley, R.S. , Jomelli, V. , Geirsdottir, A. , Kaufman, D.S. , Koch, J. et al. (2016) Glacier fluctuations during the past 2000 years. Quaternary Science Reviews, 149, 61–90. Available from: 10.1016/j.quascirev.2016.04.008

[esp5371-bib-0067] Tomkins, M.D. , Dortch, J.M. , Hughes, P.D. , Huck, J.J. , Pallàs, R. , Rodés, Á. et al. (2021) Moraine crest or slope: An analysis of the effects of boulder position on cosmogenic exposure age. Earth and Planetary Science Letters, 570, 117092. Available from: 10.1016/j.epsl.2021.117092

[esp5371-bib-0068] Vacco, D.A. , Alley, R.B. & Pollard, D. (2009) Modeling dependence of moraine deposition on climate history: the effect of seasonality. Quaternary Science Reviews, 28(7‐8), 639–646. Available from: 10.1016/j.quascirev.2008.04.018

[esp5371-bib-0069] Višnjević, V. , Herman, F. & Podladchikov, Y. (2018) Reconstructing spatially variable mass balances from past ice extents by inverse modeling. Journal of Glaciology, 64(248), 957–968. Available from: 10.1017/jog.2018.82

[esp5371-bib-0070] Whipple, K.X. , Kirby, E. & Brocklehurst, S.H. (1999) Geomorphic limits to climate‐induced increases in topographic relief. Nature, 401(6748), 39–43. Available from: 10.1038/43375

[esp5371-bib-0071] Zhang, J.‐F. , Xu, B. , Turner, F. , Zhou, L. , Gao, P. , Lü, X. & Nesje, A. (2017) Long‐term glacier melt fluctuations over the past 2500 yr in monsoonal High Asia revealed by radiocarbon‐dated lacustrine pollen concentrates. Geology, 45(4), 359–362. Available from: 10.1130/G38690.1

[esp5371-bib-0072] Zoet, L.K. & Iverson, N.R. (2020) A slip law for glaciers on deformable beds. Science, 368(6486), 76–78. Available from: 10.1126/science.aaz1183 32241945

